# Taxpayers’ Share of US Prescription Drug and Insulin Costs: a Cross-Sectional Study

**DOI:** 10.1007/s11606-024-09032-x

**Published:** 2024-10-24

**Authors:** Elizabeth Schrier, David U. Himmelstein, Adam Gaffney, Danny McCormick, Steffie Woolhandler

**Affiliations:** 1 Internal Medicine Residency Program, Department of Medicine, University of California, San Francisco, CA USA; 2 City University of New York at Hunter College, New York, NY USA; 3 Department of Medicine, Cambridge Health Alliance, Harvard Medical School, Cambridge, MA USA

**Keywords:** costs and spending, prescription drugs, insurance coverage and benefits, Medicare, Medicaid, private health insurance, diabetes

## Abstract

**Background:**

Drug prices affect government budgets directly through spending on public programs like Medicare and Medicaid, and indirectly via private coverage for public employees and tax subsidies for private insurance. Yet, the Senate parliamentarian ruled that the Senate could not use streamlined Budget Reconciliation to extend the Inflation Reduction Act’s controls on insulin co-payment or drug prices to private insurers on the grounds that their expenditures do not affect the federal budget.

**Objective:**

To quantify insulin and other drug costs borne by federal, state, and local governments, including direct expenditures and indirect government subsidies that flow through private insurers.

**Design:**

Cross-sectional analysis of expenditures for outpatient retail prescription drugs reported by respondents and their pharmacies in the 2019 Medical Expenditure Panel Survey (adjusted downward for drug rebates), supplemented with information on employment-related insurance from the US Office of Management and Budget and other sources.

**Participants:**

The civilian non-institutionalized US population.

**Main Measures:**

Direct (payments by public health insurance programs) and indirect (taxpayer-funded payments via private insurers) government expenditures for outpatient retail drugs.

**Key Results:**

Direct government expenditures for outpatient retail prescription drugs totaled $154.85 billion in 2019, including $15.68 billion for insulin. Indirect government expenditures channeled through private insurers totaled $53.59 billion (including $5.48 billion for insulins). Those indirect expenditures encompassed $32.32 billion in tax subsidies for employer-sponsored private coverage, $25 million for subsidies to private Affordable Care Act marketplace plans, and $21.24 billion for government-paid premiums for public employees and retirees. Overall, government expenditures for outpatient retail prescription drugs totaled $208.44 billion, 58.76% of all-payer spending and 65.96% of spending for insulin.

**Conclusions:**

Governments directly or indirectly fund most drug purchases, including substantial expenditures that flow through private insurers. Hence, prices paid by private insurers impact government budgets, supporting the view that government should be allowed to regulate drug prices.

**Supplementary Information:**

The online version contains supplementary material available at 10.1007/s11606-024-09032-x.

## INTRODUCTION

High drug prices and costs have triggered widespread concern. Nearly one-third of American adults report that they have failed to take a medication as prescribed due to cost within the past 12 months and 83% favor government negotiating drug prices for both Medicare and private insurers.^1^

The hardships imposed by high and escalating insulin prices have drawn particular attention; 7.6 million Americans use insulin to manage their diabetes.^2^ Medicare Part D plans alone spent $13.3 billion on insulin in 2017, up from $1.4 billion in 2007.^3^ Meanwhile, list prices for insulin—which are reflected in out-of-pocket (OOP) costs for the uninsured and for those with insurance before they meet their annual deductible—increased more than 250% between 2007 and 2018.^4^ High OOP costs contribute to medication non-adherence and insulin rationing, and even, reportedly, to several deaths.^5–7^

In response to public concern, several bills proposing caps on OOP costs for insulin have been introduced since 2021, including the Elijah E. Cummings Lower Drug Costs Now Act, the Affordable Insulin Now Act, and the Build Back Better Act. The Inflation Reduction Act (IRA) imposed penalties on drug firms that raised prices faster than inflation, a $35/month cap on OOP insulin costs for patients, and negotiations over the prices Medicare pays for some drugs. Initial versions of the IRA would have applied the penalties and caps to prescriptions for patients covered by private insurance as well as those with Medicare. However, the Senate parliamentarian ruled that list price and OOP spending limits for the privately insured would not affect the federal budget, precluding passage under the Senate’s budget reconciliation process that allows passage with a simple (rather than the usual 60%) majority vote. As a result, those provisions were stripped from the final bill.^8,9^

The parliamentarian’s ruling could affect future Congressional efforts to regulate drug prices and other costs paid by private insurance. Yet, such costs might impact the federal budget in several ways. The federal government purchases private coverage for millions of federal workers, military families, and retirees. Additionally, it subsidizes private coverage through the Affordable Care Act exchanges and provides tax subsidies that defray a substantial portion of the costs of employer-sponsored private coverage for private-sector workers. Finally, although state and local government expenditures for prescription drugs do not affect the federal budget, and hence are not relevant to the parliamentarian’s ruling, estimates of such expenditures may inform state-level efforts to address drug costs.

We sought to comprehensively assess the extent of taxpayer spending for insulin and for all outpatient retail prescription drugs in 2019.

## DATA AND METHODS

We analyzed data on insurance coverage, type of employer, and payments for prescription medications from the 2019 Medical Expenditure Panel Survey (MEPS) Full-Year Consolidated Data File and Prescribed Medicines File. The MEPS, described in detail elsewhere,^10^ is a nationally representative survey that collects detailed information on health care coverage, health care use, and expenditures by and for the civilian, non-institutionalized US population.

MEPS queries respondents about prescription fills (including refills) and obtains information on the amounts paid and sources of payment from their pharmacies (including mail order pharmacies). The sources of payments include those made by patients OOP, as well as insurance payments from both private and public insurers. We identified all filled prescriptions, which MEPS categorized according to the Multum Lexicon Therapeutic Classification Scheme.^11^ We further subcategorized insulins according to duration of effect/rapidity of onset and examined individual insulin types.

We determined mean payments per prescription for each drug category, as well as for the insulin subcategories. Because MEPS data represents payments to pharmacies, it does not account for rebates that manufacturers often pay to insurers. Hence, we also report figures adjusted downward based on a published estimate of that rebates averaged 21% overall, including 22% for Medicare Part D, 51% for Medicaid, 12% for all private insurers, and 21% for “other” federal and state payers.^12^ We assumed that no rebates are paid to the Veterans Health Administration (VHA). For a sensitivity analysis, we calculated rebates assuming 27% rebates for all non-VHA payers, based on a detailed study of rebates in Colorado in 2019.^13^

**Figure 1:**
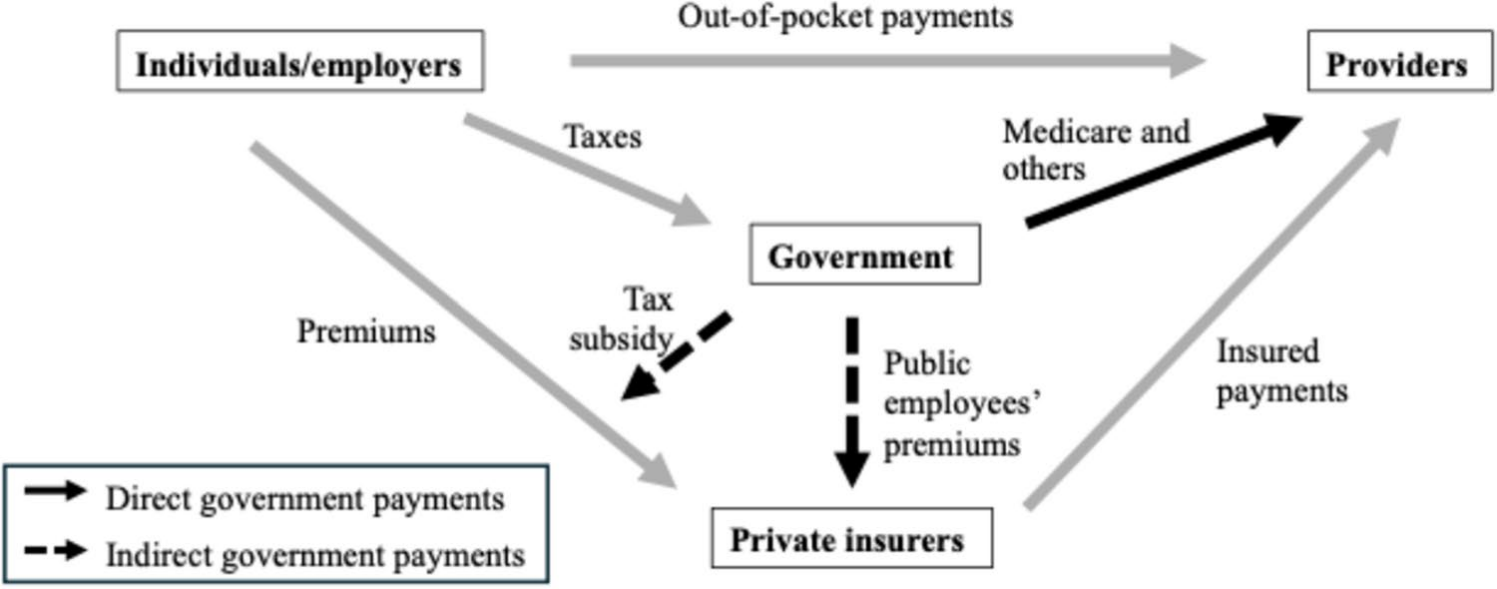
Conceptual model of flows of payments from individuals, employers, private insurers, and government to pharmacies for outpatient retail prescription drugs.

Figure 1 displays the conceptual model of the flow of funds for outpatient retail prescription drug purchases underlying our analysis. Table 1 summarizes the data sources and calculations used in our analysis.

**Table 1:** Sources of Data and Methods Used to Estimate Government Expenditures for Outpatient Retail Prescription Drugs

Payer category	Data source	Calculation
Direct government payments		
Medicare	MEPS*	Sum of Medicare-paid expenditures, with and without rebates
Medicaid	MEPS*	Sum of Medicaid-paid expenditures, with and without rebates
VA	MEPS*	Sum of VA-paid expenditures, assumes no rebates
Other federal	MEPS*	Sum of expenditures paid by “Other Federal Programs,” with and without rebates
Other state/local	MEPS*	Sum of expenditures paid by “Other State and Local Payer,” with and without rebates
Subtotal of direct government payments		Sum of categories above
Indirect government expenditures through taxpayer contributions to private insurance payments		
Tax subsidies for private insurance		
Employer-sponsored insurance	MEPS*; Office of Management and Budget (total federal tax subsidy), Quarterly Survey of State and Local taxes (ratio of state:federal income tax receipts), and NHEA (government employers’ share of employer payments for private coverage)	[Sum of payments by employer-paid private insurance] × [private employers’ share of all employer-paid private insurance] × [(federal + state and local tax subsidies)/total employer payments for employees’ premiums]
ACA exchange plans	MEPS*; CMS Health Insurance Exchange 2019 Open Enrollment Report	Sum of expenditures paid by ACA exchange plans × share of ACA exchange premiums paid by government
Government expenditures for public employees’ and retirees’ private health insurance		
Federal employees	MEPS*; Office of Personnel Management	Sum of private insurance expenditures on behalf of federal employees and dependents × share of premiums paid by federal government
State/local employees	MEPS*; Agency for Healthcare Research and Quality	Sum of private insurance expenditures on behalf of state and local government employees × share of premiums paid by state and local governments
Civilian TRICARE enrollees	MEPS*; US Government Accountability Office	Sum of TRICARE expenditures on behalf of civilians × share of TRICARE premiums paid by federal government
Subtotal of indirect government expenditures		Sum of 5 categories above

See text for rebate percentages incorporated in rebate-adjusted figures

*NHEA* National Health Expenditure Accounts

*Authors’ analysis of data from 2019 Medical Expenditure Panel Survey

Our estimates encompass direct as well as indirect taxpayer spending in 2019 for all outpatient retail drugs (i.e., excluding those administered in hospitals, clinics, or physicians’ offices, which are not reported in the MEPS data) combined and for insulins. To calculate direct spending by government health programs, we summed payments to pharmacies by Medicare, Medicaid, the Veterans Health Administration, and smaller federal, state, and local government programs.

We assessed several types of indirect government payments for medications, i.e., taxpayer-funded payments that flow through private insurers. First, to calculate expenditures for civilian government workers (and their dependents) covered by employer-based private insurance, we identified such respondents based on the MEPS variable indicating employer type. We summed private insurers’ payments for drugs on their behalf and deflated these figures by the percentage of premiums paid by workers themselves: 11% for federal workers and their families (based on US Office of Personnel Management figures on the proportion of family and individual coverage, and the weighted average of federal employees’ share of private coverage) and 21% for state and local government employees and their families (based on figures from the Agency for Healthcare Research and Quality).^14,15^

We used similar methods to estimate the federal government’s 88% share of drug costs paid by the Department of Defense’s TRICARE program on behalf of US-based civilians, e.g., dependents of military personnel and military retirees.^16^

To estimate indirect tax expenditures due to tax subsidies for workers with employment-based coverage, we calculated tax subsidies’ share of premiums paid to private insurers using previously published methods.^17,18^ We first obtained the Office of Management and Budget’s (OMB) 2019 estimates of the value of the federal income tax and payroll tax subsidies (which the OMB labels “tax expenditures”) to health care and health insurance.^19^ While state/local governments that impose income taxes also indirectly subsidize employment-based insurance through taxes, no published estimates of these subsidies are available. Hence, we estimated state and local income tax subsidies to health insurance premiums in 2019 by multiplying the value of the federal income tax subsidy by the ratio of (local + state) income tax receipts to federal income tax receipts. We calculated this ratio using data from the Census Bureau’s quarterly surveys of state and local taxes.^20^ Together, the value of federal and state tax subsidies is equivalent to 24.52% of expenditures for employer-sponsored private coverage.

To avoid double-counting tax subsidies received by government employees, we adjusted the tax subsidy estimates downward based on the share of employer-sponsored coverage paid by state/local and federal government employers reported in the National Health Expenditure Accounts.^21^

Finally, we used CMS figures on Advanced Payment Tax Credits for private coverage purchased through the Affordable Care Act (ACA) Marketplace Health Insurance Exchanges to estimate the proportion (76.6%) of exchange coverage (and hence payments for prescription drugs) attributable to public funds.^22^

To account for prescription drug rebates, we adjusted all figures downward by rebate amounts, as noted above.

All analyses used Stata version 17 procedures that account for the survey’s complex design and MEPS-provided weights to derive nationally representative estimates. The authors’ IRB does not consider analyses of de-identified, public-use data to be human subjects research.

## RESULTS

Of MEPS participants, 16,856 filled 289,675 prescriptions (weighted *n* = 3,085,807,573), including 5741 (weighted *n* = 56,237,258) for insulins. Among the 16 categories of drugs, cardiovascular agents accounted for the largest share (20.3%) of prescriptions, but only 5.4% of total payments (Table 2). While metabolic agents (including insulins) accounted for a somewhat smaller share of outpatient prescriptions (15.3%), they represented 21.2% of all payments, nearly twice the payment share of the second costliest category—central nervous system agents.

**Table 2:** Outpatient Retail Prescription Drugs by Drug Category, Mean Costs per Prescription Fill, and Percent of Total Prescription Fills and Costs, in 2019

Drug category	All drug prescriptions by category (unweighted *n* = 289,675)			
	Mean payment per prescription		% of prescriptions	% of payments
	Unadjusted*	Adjusted^†^		
Metabolic agents (including insulins)	$202	$160	15.3	21.2
Miscellaneous and unclassified agents	$613	$484	3.4	14.1
Central nervous system agents	$91	$72	18.2	11.3
Immunologic agents	$3047	$2407	0.3	7.1
Antineoplastics	$1388	$1097	0.7	6.8
Respiratory agents	$153	$121	6.3	6.7
Anti-infectives	$193	$152	4.7	6.2
Cardiovascular agents	$38	$30	20.3	5.4
Coagulation modifiers	$346	$273	2.0	4.8
Psychotherapeutic agents	$84	$66	8.3	4.8
Hormones/hormone modifiers	$68	$54	7.8	3.7
Topical agents	$116	$92	4.6	3.6
Gastrointestinal agents	$86	$68	4.8	2.8
Genitourinary tract agents	$249	$197	0.5	2.8
Nutritional products	$33	$26	2.7	0.6
Alternative medicines	$75	$59	0.2	0.1

Source: Authors’ calculations from the 2019 MEPS. All figures are weighted to be nationally representative

*Average of amounts paid by individual respondents and their insurers

^†^Adjusted downward by 21% to account for estimated prescription drug rebates for all payers combined

Per-prescription-fill costs were highest for immunologic agents ($2407 adjusted for rebates) and antineoplastics ($1097 adjusted for rebates) (Table 2). As a result, although each of these categories accounted for fewer than 1% of prescription fills, each represented about 7% of prescription drug costs.

Table 3 displays figures on the frequency and cost of insulin prescription fills according to type of insulin. Adjusted cost per insulin prescription averaged $570 (unadjusted $722), which differed minimally across major payors (data not shown). Long-acting insulins were 43.6% of insulin prescriptions. Novolog, Humalog, and Lantus together accounted for more than half of all fills and costs. Per-fill costs were highest for Novo Nordisk’s Fiasp, Novolog (both of which are brand name versions of Aspart), and Tresiba, and were lowest for generic Aspart (also made by Novo Nordisk).

**Table 3:** Outpatient Retail Insulin Prescriptions by Insulin Type, Names, Manufacturer, Mean Cost per Prescription Fill, and Percentage of Total Insulin Prescription Fills and Costs, in 2019

Type	Insulin prescriptions by type (unweighted *n* = 5741)						
	Classification						
	Generic name	Product name*	Manufacturer	Mean payment per prescription fill		% of insulin prescriptions	% total insulin payments
				Unadjusted^†^	Adjusted^‡^		
Rapid acting Short/intermediate Acting	Aspart	Novolog	Novo Nordisk	$1089	$860	17.1	25.2
		Novo FlexPen	Novo Nordisk	$356	$281	0.6	0.3
		Fiasp	Novo Nordisk	$1253	$990	0.2	0.0
		Aspart	Novo Nordisk	$67	$53	0.2	0.0
	Lispro	Humalog	Eli Lilly	$816	$645	15.2	16.6
		Admelog	Sanofi-Aventis	$233	$184	1.7	2.2
Short/intermediate acting Long acting	Regular insulin/NPH	Humulin R/N	Eli Lilly	$914	$722	5.2	6.6
		Novolin R/N	Novo Nordisk	$271	$214	4.5	1.8
Long acting Ultralong acting	Glargine	Lantus	Sanofi-Aventis	$565	$446	20.7	15.5
		Basaglar	Eli Lilly	$380	$300	9.0	5.3
		Toujeo	Sanofi-Aventis	$902	$713	3.4	4.6
		Glargine	Eli Lilly/Sanofi-Aventis	$201	$159	0.7	0.3
		Solostar	Sanofi-Aventis	$245	$194	0.3	0.1
	Detemir	Levemir	Novo Nordisk	$680	$537	9.9	9.3
Ultralong acting	Degludec	Tresiba	Novo Nordisk	$1088	$860	6.5	10.1
Unknown	Insulin, misc	N/A	N/A	$456	$360	0.5	3.1

Source: Authors’ calculations from the 2019 MEPS. Figures are weighted to be nationally representative

*Name reported in MEPS data file

^†^Average of amounts that individuals and their insurers paid per prescription fill

^‡^Adjusted downward by 21% to account for estimated prescription drug rebates for all payers combined

Prescription drug payments to pharmacies totaled $449.00 billion, equivalent to $354.71 billion after adjustment for rebates (Table 4). Payments for insulins totaled $40.60 billion unadjusted and $32.07 billion adjusted, 9.04% of the payments for all prescription medications. A sensitivity analysis using 2019 Colorado rebate figures yielded an adjusted estimate of $327.77 billion for total outpatient drug expenditures and $29.64 billion for insulins (Supplementary Table).

**Table 4:** Taxpayers’ Contributions to Retail Prescription Payments for All Drugs and for Insulins, in 2019

	All prescriptions (unweighted *n* = 289,675)			Insulin (unweighted *n* = 5741)		
	Unadjusted (millions of dollars)	Adjusted for rebates (millions of dollars)	% of total	Unadjusted (millions of dollars)	Adjusted for rebates (millions of dollars)	% of total
Total payments for prescriptions	$449,000	$354,710	100	$40,600	$32,074	100
Direct government payments						
Medicare Part D	$157,000	$122,460	34.52	$16,500	$12,870	40.13
Medicaid	$46,800	$22,932	6.46	$4150	$2034	6.34
VA	$5390	$5390	1.52	$330	$330	1.03
Other federal	$2520	$1991	0.56	$321	$254	0.79
Other state/local	$2630	$2078	0.59	$242	$191	0.60
Subtotal of direct government payments	$214,340	$154,851	43.66	$21,543	$15,679	48.88
Indirect government expenditures through taxpayer contributions to private insurance payments						
Tax subsidies for private insurance						
Employer-sponsored insurance	$36,732	$32,324	9.11	$3752	$3302	10.29
ACA exchange plans 12% rebate	$28	$25	0.01	$0.44	$0.38	0.00
Government expenditures for public employees’ and retirees’ private health insurance						
Federal employees	$2265	$1993	0.56	$81.44	$71.67	0.22
State/local employees	$15,808	$13,911	3.92	$1904	$1676	5.22
Civilian TRICARE enrollees	$6060	$5333	1.50	$489	$430	1.34
Subtotal of indirect government expenditures	$60,893	$53,586	15.11	$6227	$5480	17.08
Total tax-financed expenditures	$275,233	$208,437	58.76	$27,770	$21,159	65.96

Source: Authors calculations from the 2019 MEPS. All figures are weighted to be nationally representative. Figures for total payments, other federal, and other state are adjusted downward by 21% to account for estimated rebates. Figures for Medicare are adjusted downward by 22% for rebates. Figures for Medicaid are adjusted downward by 51% for rebates. Figures for all indirect government expenditures are adjusted downward by 12%, the average rebate for private insurers

Taxpayers’ share of payments for all outpatient retail drugs totaled 58.76% (Table 4). Direct government payments by Medicare, Medicaid, the VHA, and other government programs accounted for 43.66% of all payments. Indirect payments by federal, state, and local governments that flowed through private insurers totaled $53.59 billion adjusted for rebates ($60.89 billion unadjusted), accounting for 15.11% of total outpatient retail drug expenditures, including 9.11% for tax subsidies to employer-sponsored coverage, and 5.98% for taxpayers’ share of public employees’ and TRICARE premiums. The sensitivity analysis using 2019 Colorado rebate figures (Supplementary Table) yielded estimates that taxpayers’ expenditures for outpatient retail drugs totaled $200.92 billion, 61.30% of total expenditures for such drugs.

Taxpayers’ share of insulin costs amounted to $21.16 billion adjusted for rebates ($27.77 billion unadjusted), 65.96% of total expenditures for insulin, somewhat larger than taxpayers’ share of payments for other prescription drugs. Direct federal, state, and local government payments accounted for 48.88% of insulin spending, and indirect payments for 17.08% ($6.23 billion unadjusted, $5.48 billion adjusted for rebates), 10.29% for tax subsidies, and 6.78% for taxpayers’ share of premium costs for public employees and TRICARE.

## DISCUSSION

Taxpayers pay for 58.76% of all outpatient retail prescription drugs and almost two-thirds of insulin purchases. These figures are considerably higher than those reported in the usual tabulations of sources of payment for medications. Those tabulations reflect who “wrote the check” to the pharmacy, rather than who ultimately picked up the bill; payments made by private insurers on behalf of a government employee are labeled “private,” even if a government entity paid the entire premium. Similarly, tax subsidies to employer-sponsored coverage are not counted in National Health Expenditure Accounts tabulations,^21^ or estimates by Health and Human Services’ (HHS) Inspector General.^23^

Although health policy experts (and both the OMB and Congressional Budget Office (CBO)) are aware of these indirect sources of government payments for care, others (apparently including the Senate Parliamentarian) often fail to take account of them. A CBO document prepared after the Parliamentarian’s ruling estimated that savings for commercial insurers from provisions that remained in the IRA would reduce federal deficits by $2 billion over a decade.^24^ However, we could identify no official estimates tabulating potential government savings from the more broadly applicable price reductions in the bill’s original version, or of government expenditures—including indirect expenditures that flow through private insurers—for prescriptions drugs.

Prior studies have estimated individuals’ out-of-pocket spending for insulin and costs paid by private insurers and Medicare,^1,25^ but none have assessed overall government spending for insulin. The HHS Office of Inspector General estimated that HHS programs accounted for 41% of prescription drug funding in 2019 (approximately $151 billion).^26^ This estimate is similar to our adjusted estimate of direct government spending through such programs after adjusting for rebates, but does not include the additional $53.59 billion we estimate flows through indirect sources.

Hence, the full budgetary impact of high prices for insulin and other drugs, and potential savings for government from price controls or negotiations have been obscured. Our figures suggest that reducing US insulin prices to the Canadian or UK levels—respectively 88% and 92% lower than the US^27^—would have saved US taxpayers as much as $19.5 billion in 2019 (92% of our calculation of taxpayer payments for insulin).

Our analysis has several limitations. No comprehensive and reliable figures are available on rebates paid to insurers. Hence, our figures for net costs rely on published estimates of such rebates. Our primary analysis using payer-specific estimates of rebates yielded estimates of taxpayer payments for medications slightly lower than the more recent Colorado-based estimate of the all-payer average rebate. Other sources using varying methods suggest somewhat higher rebates,^28^ and rebate levels have likely increased since 2019. However, while calculations assuming higher rebates would decrease our dollar estimates of government expenditures, our estimates of the fraction paid by governments would not be greatly affected since both numerators and denominators would decrease. Furthermore, rebate prices are estimated to increase in tandem with list prices, often at a slightly lower rate.^29^

MEPS’ exclusion of persons in institutions, where public payment predominates, likely leads us to underestimate public payments, particularly for Medicaid and Medicare coverage of nursing home residents. However, it is reassuring that our rebate-adjustment yielded estimates of outpatient prescription drug expenditures overall, and by Medicare and Medicaid, that are similar to those provided by the National Health Expenditures Accounts.

Because our estimates encompass only outpatient prescription medications, they exclude payments for drugs administered in hospitals and physicians’ offices—which account for about 30% of total prescription drug expenditures,^30^ $37.26 billion in 2019^31^—causing us to substantially understate total government drug expenditures. Moreover, increases in prescription drug expenditures since 2019 have certainly increased government expenditures, although their effect on government’s share of expenditures is uncertain. Our tax subsidy estimates rely on simplifying assumptions regarding similarities between public- and private-sector workers with job-based coverage, e.g., that public and private sector employees are in similar tax brackets and live in states with similar average income tax rates. We also assume, as does the CBO,^24^ that reductions in drug prices would cause premium reductions for government purchasers of private coverage, and that government payers’ share of commercial premiums applies to drug purchases.

## CONCLUSION

High drug costs affect all American taxpayers, not just patients who need medications. The Inflation Reduction Act, which imposed some Medicare drug price controls, may prove a useful first step, but broader measures are needed to make drugs affordable for all patients and for government budgets.

## Supplementary Information

Below is the link to the electronic supplementary material.

Supplementary file (PDF 288 KB)
