# Supplementary material for: Taxpayers’ Share of US Prescription Drug and Insulin Costs: a Cross-Sectional Study
Source: J Gen Intern Med. 2024 Oct 24;40(6):1325–32. doi: 10.1007/s11606-024-09032-x (PMC12045909; doi:10.1007/s11606-024-09032-x)
Supplement: Supplementary file 1 — Supplementary file (PDF 288 KB) [file 11606_2024_9032_MOESM1_ESM.pdf]

Supplemental Table: Sensitivity analysis: Taxpayers' contributions to retail prescription payments for all drugs and for insulins, 2019 using 2019 Colorado estimate of rebates

	All Prescriptions (unweighted n=289,675)			Insulin (unweighted n=5,741)		
	**Unadjusted (millions of dollars)**	**Adjusted for rebates (millions of dollars)**	**% of total**	**Unadjusted (millions of dollars)**	**Adjusted for rebates (millions of dollars)**	**% of total**
**Total payments for prescriptions**	$449,000	$327,770	100.%	$40,600	$29,638	100%
**Direct government payments**						
Medicare Part D	$157,000	$114,610	34.97%	$16,500	$12,045	40.64%
Medicaid	$46,800	$34,164	10.42%	$4,150	$3,030	10.22%
VA	$5,390	$3,935	1.20%	$330	$241	0.81%
Other Federal	$2,520	$1,840	0.56%	$321	$234	0.79%
Other State/Local	$2,630	$1,920	0.59%	$242	$177	0.60%
**Subtotal – direct government payments**	$214,340	$156,468	47.74%	$21,543	$15,726	53.06%
**Indirect government expenditures through taxpayer contributions to private insurance payments**						
**Tax subsidies for private insurance**						
Employer-sponsored insurance	$36,732	$26,814	8.18%	$3,752	$2,739	9.24%
ACA exchange plans	$28	$20.44	0.01%	$0.44	$0.32	0.00%
**Government expenditures for public employees' and retirees' private health insurance**						
Federal employees	$2,265	$1,653	0.50%	$81.44	$59.45	0.20%
State/local employees	$15,808	$11,540	3.52%	$1,904	$1,390	4.69%
Civilian TRICARE enrollees	$6,060	$4,424	1.35%	$489	$357	1.20%
**Subtotal – indirect government expenditures**	$60,893	$44,452	13.56%	$6,227	$4,546	15.34%
**Total tax-financed expenditures**	**$275,233**	**$200,920.**	**61.30%**	**$27,770**	**$20,272**	**68.40%**

Source: Authors calculations from the 2019 Medical Expenditure Panel Survey (MEPS).
Notes:
All figures are weighted to be nationally representative
Figures for payments are all adjusted downward by 27% to account for estimated rebates.
